# CASE REPORT Superior Gluteal Artery Perforator Flap Breast Reconstruction Salvage Following Late Venous Congestion After Discharge

**Published:** 2010-10-13

**Authors:** Rodney K. Chan, Jon A. Mathy, Wojitec Przylecki, Lifei Guo, Stephanie A. Caterson

**Affiliations:** ^a^US Army Institute of Surgical Research, Brook Army Medical Center, San Antonio, TX 78234; ^b^Plastic and Reconstructive Surgical Unit, Middlemore Hospital, Auckland, New Zealand; ^c^Division of Plastic Surgery, Department of Surgery, Brigham and Women's Hospital, Boston, MA 02445

## Abstract

**Objective:** Microvascular thrombosis is a dreaded complication of free tissue transfer, especially in breast reconstruction. Failure often leads to complete loss of the reconstruction and affects the patient both physically and psychologically. Fortunately, most vascular compromises occur early (within 24–36 hours) while the patient is still in the hospital and intervention takes place prior to irreversible thrombosis of the microvasculature. However, failures beyond 96 hours generally have dismal prognosis, especially because the patient is already home. **Methods:** A case of successful salvage is reported after an uncomplicated superior gluteal artery perforator flap performed for breast reconstruction returned from home with thrombosis of the venous pedicle the morning of postoperative day 5. **Results:** The pedicle was promptly explored and the venous patency reestablished using a combination of mechanical and chemical thrombolysis. At her 2-year follow-up, there was no evidence of fat necrosis and a satisfactory aesthetic outcome was achieved. **Conclusion:** Late salvage of failing free flap breast reconstruction from home is possible. Educating the patient on importance of self-examination is critical to salvage. The hospital system also needs to have the resources to handle such emergencies in order for rapid operative mobilization to expedite the patient's care.

Perforator flap breast reconstructions are excellent autologous options, especially for young and active patients with a history of radiation. One drawback, however, is the inherent technical complexity associated with any free tissue transfer. Venous thrombosis accounts for the most common cause of flap loss. The timing of this event drops off exponentially, with the highest likelihood being in the first 48 hours. The possibility of thrombosed flap salvage, unfortunately, also diminishes quickly with only rare recoveries after 3 days. Here we describe successful salvage of a late flap failure from venous congestion, presenting from home. We review the timing of free flap failures from venous causes as well as reasons for successful salvage in this particular case.

## CASE REPORT

**Figure 1:**
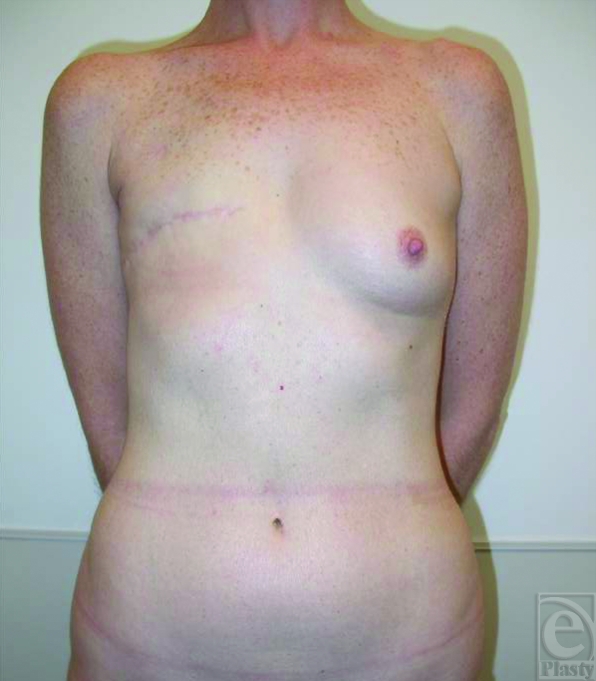
Pre-operative photograph.

The patient is an athletic 41-year-old woman who underwent delayed autologous superior gluteal artery perforator (SGAP) breast reconstruction. She was initially treated for invasive ductal carcinoma with right mastectomy followed by adjuvant chemotherapy and chest wall radiation. Her preoperative examination is shown (Fig 1).

**Figure 2:**
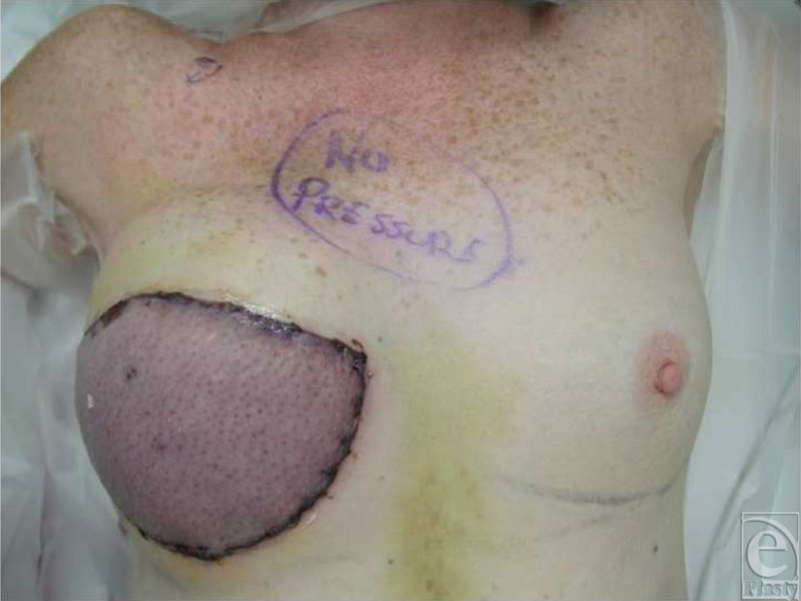
Post-operative appearance on POD 5. The SGAP flap was severely congested with rapid capillary refill.

The SGAP flap reconstruction was performed in the standard fashion.^1^ A 3.0-mm Synovis vein coupler was employed for the gluteal- to internal mammary-venous anastomosis. The patient's postoperative course was uneventful and she was discharged on postoperative day (POD) 4. On the morning of POD 5, the patient noted a new purplish discoloration to her flap, different than she had seen an hour earlier. She contacted the staff surgeon, who promptly urged her to return to the hospital. On evaluation, her flap was severely congested with rapid capillary refill (Fig 2). Doppler signal had a water-hammer quality. She was given a bolus of 5000 units of intravenous heparin and went to the operating room immediately for exploration. Her incision was made within 90 minutes of her first phone call.

Operative exposure revealed congestion of the gluteal vein with poor—but not completely absent—filling of the IMV just distal to the anastomosis. The coupler was taken down and a fresh clot was visualized within the gluteal vein that had not reached complete occlusion. Once the clot was extracted, venous drainage returned to normal and the flap decompressed instantly.

**Figure 3:**
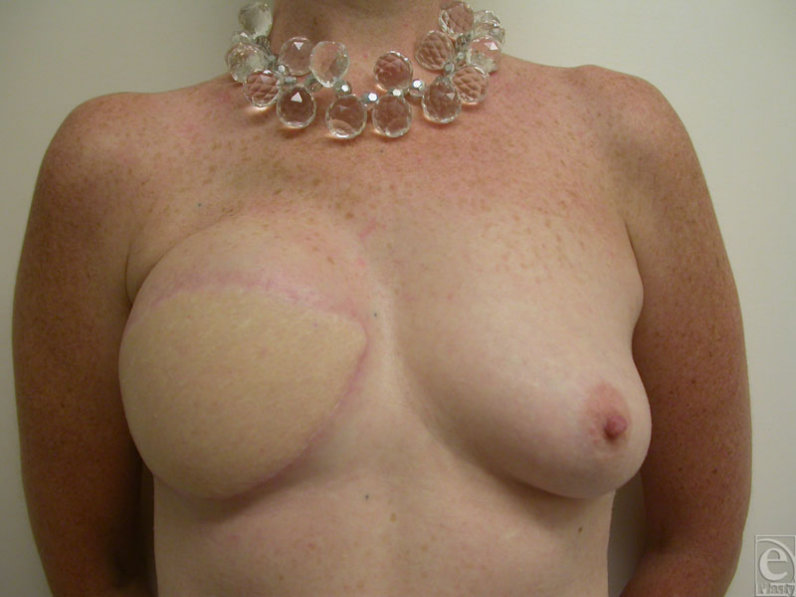
Follow up photograph at 2 years without appreciable fat necrosis.

The flap was then back-irrigated with both heparinized saline and tissue plasminogen activator. Following a short period of open drainage, normal color returned, and oxygenated blood was detected at all trimmed edges of the flap. The venous anastomosis was reperformed, again using a venous coupler, at a more proximal IMV segment of larger caliber. The flap was then reinset into the mastectomy pocket. She was anticoagulated for the remainder of her hospitalization. Subcutaneous low-molecular-weight heparin was used as an outpatient for 3 additional weeks. Her flap remains healthy and viable at long-term 2-year follow-up with no appreciable fat necrosis (Fig 3).

## DISCUSSION

Free perforator flaps are certainly the latest and most exciting advances in plastic surgery breast reconstruction. At the latest review, the number of the perforator flap breast reconstruction has dramatically increased.^2^ There are proponents to each type of autologous breast reconstruction. In addition, perforator flap breast reconstruction is not for every patient or every surgeon. In young and active patients, muscle sparing procedures such as perforator flaps arguably offer a distinct advantage.

One inherent drawback to free flap breast reconstruction, however, is that the procedure is subject to all the technical complexities and risks associated any free tissue transfer. This includes the use of specialized micro-instrumentation, operative microscope, prolonged operative time, need for intensive postoperative monitoring, and need for specialized teams. There is also an inherent risk of flap failure or compromise. Because of the potential for immediate takebacks such as in this case, the operative surgeon and team must have the resources to handle such emergencies.

Pedicle thrombosis from either arterial or venous causes is a dreaded complication, leading to complete loss of the reconstruction. Free flap loss rates fortunately are very low and even lower for superspecialized breast microsurgeons.^3^ The general microsurgical experience at the University of Texas M.D. Anderson shows a 5% flap failure rate in their series of 990 free flaps published in 1996.^4^,^5^ Since then this has been validated by numerous groups. Reasons for early flap failure within the first day are generally arterial in origin. Higher risk of outflow failure secondary to venous congestion starts from 24 hours and can extend to 7 days postoperatively with most fail within the first 2 to 3 days. After 3 days in their study, there were no flap salvages. Chen et al^6^ also confirm that rates of salvage are quite low at more than 96 hours out in their series of 1142 free flaps.^6^ While late failures have been successfully salvaged and reported in the literature, the M.D. Anderson quotes the decrease in intensive (q1h) monitoring as possible reason why they had no late salvages.

The cause for venous thrombosis is not always obvious. Technical reasons such as kinking of the pedicle, twisting of the anastomosis, and external compression such as from hematoma are reasons leading to the early development of thrombus. Recently, Davison et al^7^ reported the underrecognized incidence of hypercoagulability as a potential cause of venous thrombosis.^7^ In the case of this patient, she did not appear to have a hypercoagulable state by history and there were no obvious technical errors. Short-term mechanical compression is always possible with ipsilateral arm motion resulting in pectoralis muscle stretch near the pedicle. Patients are instructed to strictly limit arm motion with no heavy lifting, no pushing or pulling with the effected arm, and no shoulder abduction greater than 60°. Pedicle compression from pectoralis muscle contraction may be unavoidable.

Over the past decade, the length of inpatient stays, including that of free flap patients, has dropped significantly. In the initial stages of microsurgery, free flap patients would remain monitored in the hospital for weeks. Currently in our institution, patients with uncomplicated perforator flap breast reconstructions are discharged regularly on POD 4. The decision to discharge free flap patients is related to basic recovery of the patient (ability to ambulate, eat, tolerate oral narcotics, etc) but also the need for postoperative flap monitoring. Our practice is to monitor the flaps q1h for 48 hours and then q2h until discharge on POD 4. It is rare to have venous compromise at POD 5 and even rarer to salvage the flap if patient has been discharged because of inevitable time delay to operating room.

This is a successful SGAP flap salvage, presenting as a late venous obstruction from home. We credit the patient's own vigilance and fortuitous proximity to the hospital, as well as rapid operative team mobilization, which allowed us to reverse her evolving venous obstruction prior to total occlusion and flap death. The question does come up regarding whether patients should be monitored for a longer period of time, such as done routinely in European nations. Based on data from the University of Texas M.D. Anderson cancer center, monitoring 1000 patients for 4 additional days until POD 7 would potentially salvage only 2 additional flaps. This may be justifiable only if there is no other way to repair the defect should the free flap be lost; that is rarely the case with breast reconstructions although a loss perforator flap breast reconstruction is arguably less forgiven than other free flaps. For the majority of free flaps, however, close monitoring for 4 days is probably sufficient.

## CONCLUSION

Late salvage of failing free flap breast reconstruction is possible. Educating patient the importance of self-examination is critical to salvage from home. The hospital system also needs to have the resources to handle such emergencies in order for rapid operative mobilization to expedite the patient's care.
